# Desmopressin improves intestinal functional capillary density and decreases leucocyte activation in experimental endotoxemia in the rat

**DOI:** 10.1186/cc9676

**Published:** 2011-03-11

**Authors:** L Wagner, I Drzymulski, D Pavlovic, D Henzlers, M Wendt, C Lehmann

**Affiliations:** 1 Greifswald University, Greifswald, Germany; 2 Dalhousie University, Halifax, Canada

## Introduction

The vasopressin analogue desmopressin (DDAVP), a selective agonist of the vasopressin V2 receptor, is known to cause vasodilatation in addition to its haemostatic effects. To verify whether desmopressin could be beneficial in sepsis we investigated its effects on intestinal microcirculation in experimental endotoxemia in rats.

## Methods

In Lewis rats (six groups, 10 animals each) the effects of vasopressin (VAS) (0.06 U/340 g/minute) and DDAVP (1 μg/kg/ml) on the terminal ileum microcirculation 2 hours after introducing endotoxemia (5 mg/kg lipopolysaccharide (LPS), i.v.) were examined using intravital fluorescence microscopy.

## Results

**Figure 1:**
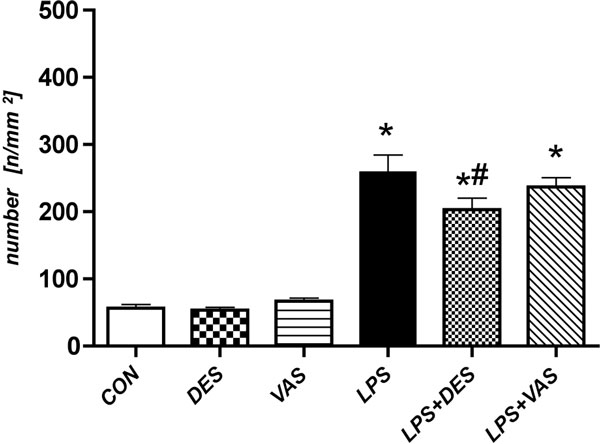
**Number of adherent leucocytes in venules (*n*/mm^2^)**. *P < 0.001 for all LPS vs. all controls; #*P *< 0.05 for LPS+DES vs. LPS.

Although desmopressin administration (DES-group) increased the number of rolling leucocytes in V3 venules (*P *< 0.05 vs. CON-group), the number of firmly adhering leucocytes in V1 venules of the LPS-group was significantly reduced (LPS-group: 259 ± 25.7 vs. LPS+DES-group: 203 ± 17.2 *n*/mm^2^; *P *< 0.05) (Figure 1). Additionally, DDAVP treatment improved impaired functional capillary density (FCD) following LPS in all examined intestinal layers (*P *< 0.001 vs. LPS-group), while the density of nonfunctional capillaries was significantly reduced (*P *< 0.001 vs. LPS-group). Vasopressin administration deteriorated FCD in endotoxemic and non-endotoxemic rats (*P *< 0.05 vs. CON-group or LPS-group). Three hours after LPS challenge, TNFα levels were reduced in both DDAVP-treated and vasopressin-treated LPS-groups (LPS-group: 429 ± 119; LPS+DES-group: 262 ± 21.9; LPS+VAS-group: 249 ± 46.5 pg/ml; *P *< 0.05).

## Conclusions

Desmopressin administration improved microvascular perfusion and reduced inflammatory response in experimental endotoxemia.

